# Enhanced heat transfer analysis on Ag-Al$_{2}$O$_{3}$/water hybrid magneto-convective nanoflow

**DOI:** 10.1186/s11671-024-03975-0

**Published:** 2024-02-22

**Authors:** M. Ragavi, T. Poornima

**Affiliations:** Department of Mathematics, School of Advanced Sciences, Vellore Institute of Technology, Vellore, India

**Keywords:** Stretching sheet, Nanoparticle shapes, MHD, Joule heating, Convective boundary conditions, Porous medium, Partial slip

## Abstract

The primary goal of this investigation is to examine the heat and flow characteristics of a hybrid nanofluid consisting of silver (Ag) and aluminum oxide (Al$$_{2}$$O$$_{3})$$ nanoparticles over an unsteady radially stretching sheet embedded in porous medium. The investigation is conducted under the influence of several key parameters, namely joule heating, viscous dissipation, porous, slip, and suction. The technique of similarity transformations is used to transform the governing system of PDEs into nonlinear ODEs and the bvp4c solver is used to solve them numerically. The present study examines the influence of sphere and platelet shape nanoparticles on the temperature and velocity profiles. The outcomes are discussed through graphs and tables. A rise in the porous, slip, and suction parameters makes the velocity profile decrease gradually. The temperature escalates when Biot number, magnetic parameter, and Eckert number increase. As compared to sphere shapes, platelet-shaped nanoparticles exhibit the greatest heat transfer and flow. Results reveal that by using Ag-Al$$_{2}$$O$$_{3}$$/H$$_{2}$$O hybrid nanofluid with a volume fraction of 5%, the heat transfer enhancement of platelet shape nanoparticles increased by 11.88% than sphere-shaped nanoparticles. Overall, the platelet shape of nanoparticles offers distinctive advantages in various engineering applications, primarily due to their large surface area, anisotropic properties, and tunable surface chemistry. These properties make them versatile tools for improving the performance of materials and systems in engineering fields. The findings can contribute to the design and optimization of nanofluid-based systems in various engineering applications, such as heat exchangers, microfluidics, and energy conversion devices.

## Introduction

In recent years, there has been significant research on the heat transfer properties of nanofluids. This is due to their potential use in a variety of fields, including electronics, thermal management, and energy harvesting. Nanofluids are suspensions of nanoparticles in a base fluid that enhance thermal conductivity. This enhancement is due to the increased surface area of the nanoparticles which allows for more efficient heat transfer. The flow of a hybrid nanofluid over a stretching sheet has been extensively studied in various research papers. By taking into account both the no-slip and velocity slip conditions, Zainal et al. [1] investigate the unsteady motion of a hybrid nanofluid via a convectively heated stretching/shrinking sheet. Hiranmoy et al. [2] explore the combined effects of unstable and heat radiation on the flow of a mixture nano liquid caused by a shrinking disk, with an emphasis on the thermophysical characteristics of the flow near a stagnation point. In this work, Cu and Al2O3 nanoparticles are combined with water to generate a hybrid nanofluid. The findings demonstrate that conventional nanofluid has a slower heat transfer rate than hybrid nanofluids, which makes it potentially beneficial for heat exchangers and electronic cooling systems. The behavior of a 2D laminar magnetohydrodynamics couple stress hybrid nanofluid flowing through a pores stretching/shrinking plate with mass transpiration and Brinkman ratio is examined by Anusha et al. [3]. Khan et al. [4] investigate entropy generation in a 2D flow of magneto Williamson hybrid nanofluid, comprising cobalt ferrite and titanium oxide nanoparticles and undergoes surface-catalyzed reactions by a thin needle. References [5–9] provides advanced heat transfer studies in nanofluid flows.

The study by Bibi and Naseem [10] provides valuable insights into axisymmetric hydromagnetized heat transfer with Joule heating and radiative effect. Swain et al. [11] conducted an analysis of the Magnetohydrodynamics (MHD) movement and gradient heat exchange of a Newtonian fluid across an extended sheet situated in a porous medium. The study encompasses various applications, including the utilization of numerous heating gadgets and their relevance in industrial operations, such as food processing, and polymer processing. Sreenivasulu et al. [12] looked at the distribution of electric resistance heating along a nonlinear stretching sheet over a three-dimensional Carreau nanoliquid while taking heat and mass transfer with nonlinear radiation and zero mass flux into account. Waini et al. [13] explore the flow of a micropolar fluid through a stretching or shrinking sheet in the presence of Al$_2$O$_3$ and Cu nanoparticles, considering the effects of viscous dissipation and Joule heating. The research is used in various industries such as manufacturing, and chemical engineering. Tarakaramu et al. [14] examined the joule heating and non-linear thermal radiation’s impact on lateral surface stretching in MHD three-dimensional viscoelastic nanofluid flow. Several studies have looked at convective heat transmission via a extended sheet. Under the impact of boundary conditions involving convection, Srisailam et al. [15] investigated the flow and heat transfer on a permeable stretched sheet of magnetohydrodynamic nanofluid. Several investigations on convective heat transport across a stretched sheet are presented in [16–20].

Numerous studies have examined the consequence of different parameters on the heat transfer and flow properties of fluids across radial stretching sheets. Azeem et al. [21] examined the heat transmision and flow characteristics of a magnetohydrodynamic viscous liquid across a nonlinear radial permeable stretched surface. The flow is generated by a non-linear stretching sheet and is impacted by continual suction or blowing of the fluid over the porous sheet. Masood et al. [22] utilized the Homotopy Analysis Technique (HAM) to analyze the flow of a non-Newtonian fluid over a sheet that is stretched radially while maintaining axisymmetry. It focuses on the axisymmetric Sisko fluid flow over a sheet and comes up with a nonlinear ordinary differential equation for the boundary layer flow. Munir et al. [23] explore the heat transmission to maintain consistent movement of a second-grade flow across a porous sheet that extends radially, considering a power law stretching of the sheet and the presence of a transverse magnetic field. This study’s results apply to both Newtonian and power-law fluids. Dianchen et al. [24] developed a mathematical model to analyze the flow of an axisymmetric steady magnetohydrodynamic (MHD) Carreau nanofluid. This study considers the impact of nonlinear thermal radiation and a chemical reaction as it flows past a surface that is radially stretched. A third-grade fluid’s flow and heat transfer across a nonlinear radially expanding sheet are investigated by Khan et al. [25]. The findings from the study enhance our comprehension of heat transfer in intricate systems, which can be applied in diverse engineering and industrial procedures.

Ahmed et al. [26] looked at the heat transmission and flow of a power law liquid model through a radially stretched sheet while an even magnetic field was supplied perpendicular to the flow direction. Shahzad et al. [27] address the boundary layer flow and heat transfer of a viscous fluid across an unstable stretched permeable surface. The implications of the relevant factors on the energy and velocity profiles was also explored. Faraz et al. [28] studied the impact of thermo-diffusion on a Casson flow that is axisymmetric and passes over a radially stretched sheet with several slip variables, as well as the force of chemical reaction. Later Faraz et al. [29] conducted a study to assess the impact of thermal radiation and mixed convection on axisymmetric Casson fluid flow under the influence of a magnetic field and nanoparticles. The study’s results could be applied to the development of magnetic nanomaterials and the high-temperature processing of magnetic nanopolymers. Natalia et al. [30]expand their study from typical impingement rotational stagnation-point movement over a radially porous elastic sheet in a viscous liquid to a water-based nanofluid.

In the presence of magnetic influences, thermal radiation, and thermal conductivity, Muhammad et al. [31] studied the impact of the morphologies of molybdenum disulfide (MoS2) nanoparticles on the rotational motion of nanofluid along an elastically expanded sheet. A study by Akinshilo [32] shows that laminar nanoparticles have higher temperatures and thermal conductivity compared to cylindrical and spherical particles when fluid moves through a porous channel. This has potential implications for energy conservation, nanofluidics, and micromixing. Tamour et al. [33] studied the effect of copper nanoparticle form on heat transmission in a 3D MHD nanofluid. They looked at a rotating flow over an expanding sheet and used Chebyshev wavelets to get numerical results. Hamza et al. [34] conducted a study to analyze how different forms of silver nanoparticles impact on enhancing heat transfer and irreversibility in a hydromagnetic water-based nano liquid flow passing through a stretched sheet that is convectively heated. The results indicate that disk-shaped nanoparticles exhibit a higher rate of heat transfer, whereas cylindrical nanoparticles lead to elevated entropy and irreversible fluid friction. Hayat et al. [35] have studied the effect of particle form on Ag-nanofluid flow and heat transmission over a stretched surface. According to the research, platelet-shaped nanoparticles had the greatest flow and heat transmission rates, both in velocity and temperature profile.

Motivated by the aforementioned literature review, the shape effects of Ag-Al$_{2}$O$_{3}$/H$_{2}$O hybrid nanofluid across an unsteady radial stretching sheet immersed in absorbent media with slip, suction, joule heating, and convective boundary conditions have not yet been addressed. Ever wondered how the unique shapes of Nanoparticles influence their flow over a radially stretching sheet. This article delves into the the revelations beneath the influence of sphere and platelet-shaped nanoparticles on the flow. The energy and momentum equations are solved using the fourth-order Runge–Kutta method along with the shooting technique. Ag-Al$_{2}$O$_{3}$/H$_{2}$O hybrid nanofluid exhibits excellent thermal conductivity and stability, making it a valuable option for heat transfer in electronics cooling and industrial processes. Silver nanoparticles have a high thermal conductivity, which can improve the heat transfer properties of the nanofluid. On the other hand, aluminum oxide nanoparticles can enhance stability and prevent agglomeration. When Ag-Al$_{2}$O$_{3}$/H$_{2}$O nanofluid circulates through the device, it absorbs excess heat and transfers it away from essential components, enhancing overall device performance and prolonging its lifetime. These findings could be used as efficient cooling agents to dissipate heat from electronic components like microprocessors, LEDs, and high-power processors. The controlled flow of hybrid nanofluid over a radially stretching sheet could enhance heat transfer and improve the uniformity of the thin film deposition process, making it applicable in industries such as electronics, optics, or surface coatings. We investigate the impact of several factors on temperature, and velocity profiles. The outcomes are shown in a table and numerous graphs.

## Mathematical model

**Fig. 1:**
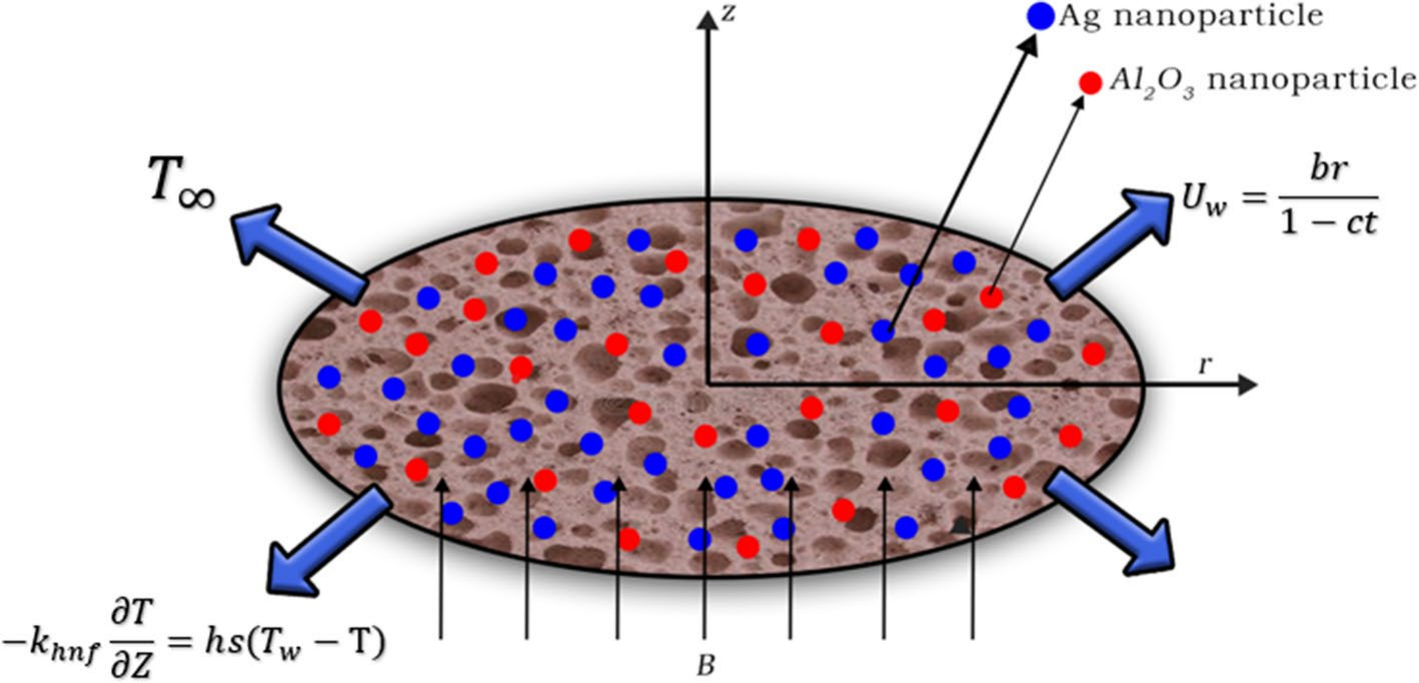
Flow geometry of stretching sheet

In this model, a unsteady 2D boundary layer Ag-Al$_{2}$O$_{3}$/H$_{2}$O hybrid nanofluid flow across a radially stretching sheet imbedded in a porous medium is evaluated with slip,suction,joule heating and viscous dissipation. The physical model under discussion is shown schematically in Fig 1. The sheet is located in the plane z=0. The radial and axial velocity components are denoted by u and w. The cylindrical polar coordinate $(r,\theta ,z)$ is used because the flow is rotationally symmetric, the physical parameters do not change with the angle $\theta$ and the velocity field is $v(r,z)=[u(r,z),0,w(r,z)]$. Flow occurs along the radial direction with velocity $U_{w}=\frac{br}{1-ct}$ and the surface temperature is $T_{w}=T_{\infty }+T_{r}\left( \frac{br^2}{2v}\right) {(1-ct)}^\frac{-3}{2}$. A consistent magnetic field $B=\frac{B_0}{\sqrt{(1-ct)}}$ is applied at a right angle to the sheet (z-direction). The stretching sheet creates a velocity gradient in the fluid, which causes the nanofluid to flow.

With the predetermined circumstances mentioned above, the boundary layer equations can be formulated as follows [35, 37, 39]:

1 $$\begin{aligned}{} & {} \frac{\partial {u}}{\partial {r}}+\frac{u}{r}+\frac{\partial {w}}{\partial {z}} =0 \end{aligned}$$

2 $$\begin{aligned}{} & {} \frac{\partial {u}}{\partial {t}}+u\frac{\partial {u}}{\partial {r}}+w\frac{\partial {u}}{\partial {z}}=\frac{\mu _{hnf}}{\rho _{hnf}}\frac{\partial ^2{u}}{\partial {z^2}}\nonumber \\{} & {} -\frac{\sigma _{hnf}}{\rho _{hnf}}B^2{(r,t)}u-\frac{\mu _{hnf}}{\rho _{hnf}{k^*}}u \end{aligned}$$

3 $$\begin{aligned}{} & {} \frac{\partial {T}}{\partial {t}}+u\frac{\partial {T}}{\partial {r}}+w\frac{\partial {T}}{\partial {z}}=\alpha _{hnf}\frac{\partial ^2{T}}{\partial {z^2}}\nonumber \\{} & {} +\frac{\mu _{hnf}}{(\rho {C_{p}})_{hnf}}{\left( \frac{\partial {u}}{\partial {z}}\right) ^2}+\frac{\sigma _{hnf}}{(\rho {C_{p}})_{hnf}}B^2{(r,t)}u^{2} \end{aligned}$$

and the boundary conditions are [28, 36]

4 $$\begin{aligned} \left. \begin{aligned} at&\hspace{0.2cm} z=0:u=U_{w}+D\frac{\partial {u}}{\partial {z}}, w=w_{0}, -k_{hnf}\frac{\partial {T}}{\partial {z}}=hs{(T_{w}-T_{\infty })} \\ as&\hspace{0.2cm} z\rightarrow \infty : u\rightarrow 0, T\rightarrow {T_{\infty }} \end{aligned} \right\} \end{aligned}$$

The thermophysical properties of *Ag* and $Al_{2}O_{3}$ nanoparticles incorporated into base fluid $H_{2}O$ are delineated as follows [38]:

5 $$\begin{aligned}{} & {} \alpha _{hnf}=\frac{k_{hnf}}{\left( \rho {C_{p}}\right) _{hnf}} \nonumber \\{} & {} \rho _{hnf}=(1-\delta _{1}-\delta _{2})\rho _{f}+\delta _{1}\rho _{s1}+\delta _{2}\rho _{s2} \nonumber \\{} & {} \mu _{hnf}=\mu _{f}{(1+w_{1}\delta _{2}+w_{2}\delta _{2}^2)} {(1+w_{1}\delta _{1}+w_{2}\delta _{1}^2)} \nonumber \\{} & {} \sigma _{hnf}=\sigma _{f}\left( 1+\frac{3(\frac{\sigma _{s2}}{\sigma _{f}}-1){\delta _{2}}}{(\frac{\sigma _{s2}}{\sigma _{f}}+2)-(\frac{\sigma _{s2}}{\sigma _{f}}-1){\delta _{2}}} \times 1+\frac{3(\frac{\sigma _{s1}}{\sigma _{f}}-1){\delta _{1}}}{(\frac{\sigma _{s1}}{\sigma _{f}}+2)-(\frac{\sigma _{s1}}{\sigma _{f}}-1){\delta _{1}}}\right) \nonumber \\{} & {} (\rho {C_{p}})_{hnf}=(1-\delta _{1}-\delta _{2})(\rho {C_{p}})_{f} \nonumber \\{} & {} +(\rho {C_{p})_{1}}\delta _{1}+(\rho {C_{p})_{2}}\delta _{2} \nonumber \\{} & {} \frac{k_{hnf}}{k_f}=\frac{k_{s2}+\left( n-1\right) k_f+\left( n-1\right) \left( k_{s2}-k_f\right) \delta _{2}}{k_{s2}+\left( n-1\right) k_f-\left( k_{s2}-k_f\right) \delta _{2}}\nonumber \\{} & {} \quad \times \frac{k_{s1}+\left( n-1\right) k_f+\left( n-1\right) \left( k_{s1}-k_f\right) \delta _{1}}{k_{s1}+\left( n-1\right) k_f-\left( k_{s1}-k_f\right) \delta _{1}} \end{aligned}$$

where $\delta _{1}$, $\delta _{2}$ denote *Ag* and $Al_{2}O_{3}$ nanoparticle volume fractions, $\alpha _{hnf}$, $\sigma _{hnf}$, $\mu _{hnf}$,$k_{hnf}$, $\rho _{hnf}$, and ${(\rho {C_{p}})_{hnf}}$ are diffusivity, electrical conductivity, dynamic viscosity, thermal conductivity, density, and heat capacity of hybrid nanofluid, respectively. The subscripts *f*, *hnf*, and *s*1, *s*2 stand for the working fluid, hybrid nanofluid, first solid particle (*Ag*), and second solid particle (Al$_{2}$O$_3$). Here $w_{1}$ and $w_{2}$ are viscosity enhancement heat capacitance coefficients, and *n* is the size of nanoparticles of various shapes.

Introducing the similarity transformations and dimensionless variables as follows [35]

6 $$\begin{aligned}{} & {} \psi =\frac{r^{2}U_{w}f(\eta )}{\sqrt{Re}}\nonumber \\{} & {} \theta {(\eta )}=\frac{T-T_{\infty }}{T_{r}{\left( \frac{br^{2}}{2v}\right) }{(1-\alpha {t})^\frac{-3}{2}}} \nonumber \\{} & {} \eta =\frac{z}{r}\sqrt{Re} \end{aligned}$$

The longitudinal and transverse components of velocity are

7 $$\begin{aligned} u= & {} -\frac{1}{r}\frac{\partial {\psi }}{\partial {z}} =U_{w}f^{'}{(\eta )} \nonumber \\ w= & {} \frac{1}{r}\frac{\partial {\psi }}{\partial {r}} =-2U_{w}Re^{\frac{-1}{2}}f{(\eta )} \end{aligned}$$

Equations (2, 3) can be transformed into a set of ordinary differential equations with the boundary condition.

8 $$\begin{aligned}{} & {} \frac{B_{1}}{B_{3}}f^{'''}-M\frac{B_{4}}{B_{3}}f^{'}-P_{k}f^{'}\nonumber \\{} & {} \quad -J{\left( \frac{\eta }{2}f^{''}+f^{'}\right) }-{(f^{'})^{2}} +2ff^{''}=0 \end{aligned}$$

9 $$\begin{aligned}{} & {} \frac{B_{2}}{B_{5}P_{r}}\theta ^{''}-{\left( \frac{J}{2}{(3\theta +\theta ^{'}{\eta})}+2f^{'}\theta -2f\theta ^{'}\right) }\nonumber \\{} & {} \quad +\frac{B_{1}}{B_{5}}E_{k}{(f^{''})^{2}}+\frac{B_{4}}{B_{5}}ME_{k}{(f^{'})^{2}}=0 \end{aligned}$$

The boundary conditions (4) become

10 $$\begin{aligned} \left. \begin{aligned} f(\eta )=F_{w}, \hspace{0.2cm}f^{'}(\eta )={(1+N_{s}f^{''}(\eta ))},\hspace{0.2cm} \theta ^{'}(\eta )=-B_{i}{(1-\theta (\eta ))} \hspace{0.4cm} at&\hspace{0.2cm} \eta =0 \\ \hspace{0.1cm} f^{'}{(\eta )}\rightarrow {0},\hspace{0.2cm} \theta {(\eta )}\rightarrow {0} \hspace{0.4cm} as&\hspace{0.2cm} \eta \rightarrow \infty \end{aligned} \right\} \end{aligned}$$

The constants $B_{1}$, $B_{2}$, $B_{3}$, $B_{4}$ and $B_{5}$ from the above equation are defined as

11 $$\begin{aligned} B_{1}=\frac{\mu _{hnf}}{\mu _{f}}, B_{2}=\frac{k_{hnf}}{k_{f}}, B_{3}=\frac{\rho _{hnf}}{\rho _{f}}, B_{4}=\frac{\sigma _{hnf}}{\sigma _{f}}, B_{5}=\frac{(\rho {C_{p}})_{hnf}}{(\rho {C_{p}})_{hnf}} \end{aligned}$$

Several constants without dimensions are as follows: $P_{k}=\frac{\mu _{f}(1-\alpha{t})}{\rho _{f}bk*}$ is the porosity parameter, $E_{k}=\frac{U_{w}^{2}}{c_{p}(T-T_{\infty })}$, is the Eckert number, $J=\frac{\alpha }{b}$ stands for the unsteadiness parameter, $P_{r}=\frac{\left( \rho {c_{p}}\right) _{f}v_{f}}{k_{f}}$ is a Prandtl number, $M=\frac{B_{0}^{2}\sigma _{f}}{b\rho _{f}}$,is the magnetic parameter, $N_{s}=D\sqrt{\frac{U_{w}v_{f}}{r}}$ refers to slip parameter, $F_{w}=-\frac{w_{0}}{2}\sqrt{\frac{1-\alpha {t}}{v_{f}}}$ is the suction parameter, $B_{i}$=$\frac{hs}{k}$ $\sqrt{\frac{v}{b}}$ is the Biot number, *Re*=$\frac{rU_{w}}{v_{f}}$ is Reynolds number.

The local Skin friction coefficient (*Cf*), and the local Nusselt number (*Nu*) are defined as follows:

12 $$\begin{aligned} C_{f}=\frac{\tau _{w}}{\rho _{f}U_{w}^{2}}, \hspace{0.2cm} Nu=\frac{rq_{w}}{k_{f}(T_{w}-T_{\infty })} \end{aligned}$$

where $\tau _{w}=\mu _{hnf}{\left( \frac{\partial {u}}{\partial {z}}\right) _{z=0}}$ shear stress on wall, $q_{w}=-k_{hnf}{\left( \frac{\partial {T}}{\partial {z}}\right) _{z=0}}$ represents wall heat flux. Equation 12 can be reduced to the following form:

13 $$\begin{aligned}{} & {} C_{f}Re^{\frac{1}{2}}={(1+w_{1}\delta _{2}+w_{2}\delta _{2}^{2})}{(1+w_{1}\delta _{1}+w_{2}\delta _{1}^{2})}f^{''} \nonumber \\{} & {} NuRe^{\frac{-1}{2}}=-\frac{k_{hnf}}{k_{f}}\theta ^{'}(0) \end{aligned}$$

The heat transfer enhancement $H_{R}$ is specified as follows [40].

14 $$\begin{aligned} H_{R}=\frac{NuRe^{\frac{-1}{2}}(Nanofluid)-NuRe^{\frac{-1}{2}}(Basefulid)}{NuRe^{\frac{-1}{2}}(Basefuild)}\times {100} \end{aligned}$$

## Solution procedure

Using MATLAB’s renowned BVP4C solver, the highly nonlinear ordinary differential Eqs. (8) to (9) subject to boundary conditions (10) are numerically solved. BVP4C is a finite difference code that implements the three-stage Lobatto III formula and it is a built in function used to estimate the numerical outcomes. First, we convert the higher-order differential equation into the first-order differential equation using the substitution *f*=q(1), $f^{'}$=q(2), $f^{''}$=q(3), $\theta$=q(4), $\theta ^{'}$=q(5). The specific procedure is as follows [41]:

$$\begin{aligned}{} & {} f^{'}=q(2) \\{} & {} f^{''}=q(3) \\{} & {} f^{'''}=\left[ \frac{MB_{4}}{B_{3}}q(2)+ P_{k}q(2)+J{\left( \frac{\eta }{2}q(3)+q(2)\right) }\right. \\{} & {} \left. +{\left( q(2)\right) }^{2}-2q(1)q(3)\right] \frac{B_{3}}{B_{1}}\\{} & {} \theta ^{''}=\left[ \frac{J}{2}\left( 3q(4)+q(5)\right) +2q(2)q(4)-2q(1)q(5) \right. \\{} & {} \left. -\frac{B_{1}}{B_{5}}E_{k}(q(3))^{2}-\frac{B_{4}}{B_{5}}ME_{k}(q(2)^{2})\right] \frac{B_{5}Pr}{B_{2}} \end{aligned}$$

and the boundary conditions are $q_{1}{(0)}=F_{w}$, $q_{2}{(0)}=[1+N_{s}q_{3}{(0)}]$, $q_{2}{(\infty )}\rightarrow {(0)}$, $q_{5}{(0)}=-B_{i}{[1-q_{4}(0)]}$, $q_{4}{(\infty )}\rightarrow {(0)}$

Missed initial conditions are obtained with the aid of shooting technique, which reduces the CPU time. Later, a finite value for $\eta$ at $\infty$ is chosen in order to satisfy the boundary conditions. Our majority of processes are conducted with different values at $\eta \rightarrow {\infty }$, which is required to understand the boundary conditions for all value of the parameters.

## Results and discussion

**Fig. 2:**
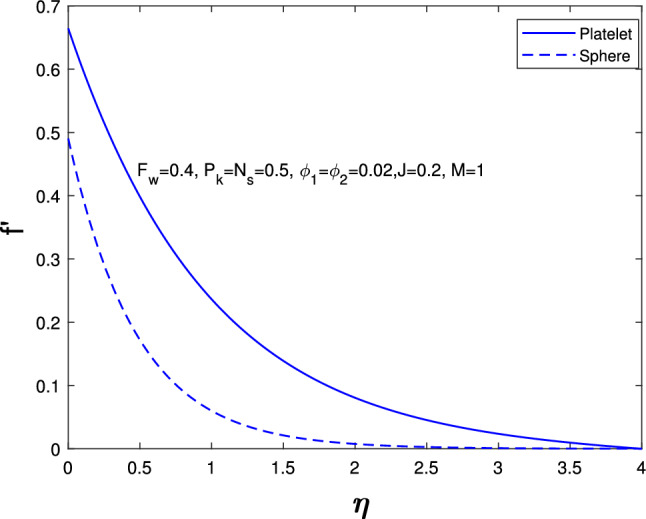
Influence in velocity distribution

**Fig. 3:**
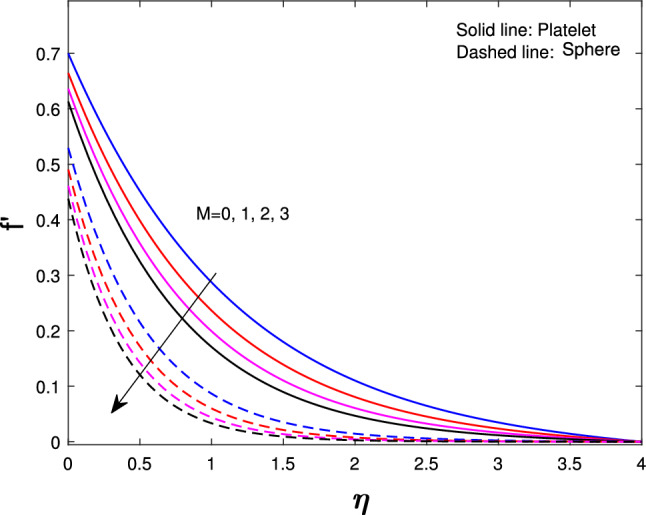
Influence of *M* on $f^{'}{(\eta )}$

**Fig. 4:**
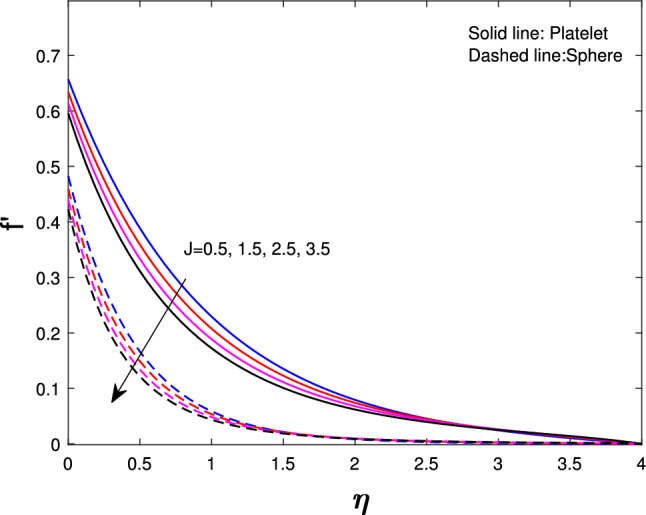
Influence of *J* on $f^{'}{(\eta )}$

**Fig. 5:**
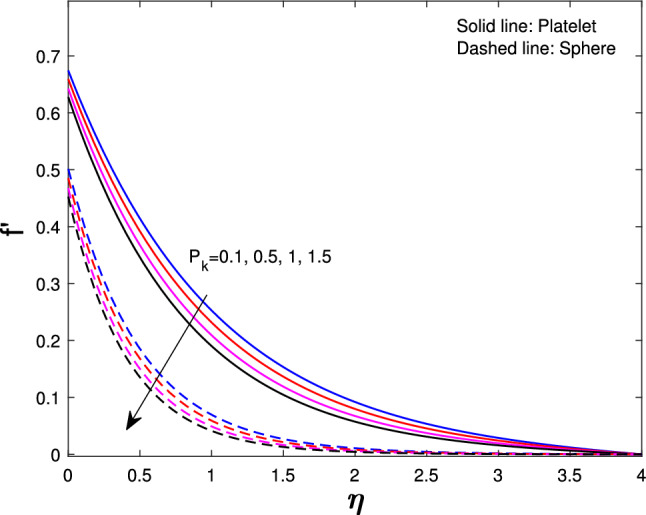
Influence of $P_{k}$ on $f^{'}{(\eta )}$

**Fig. 6:**
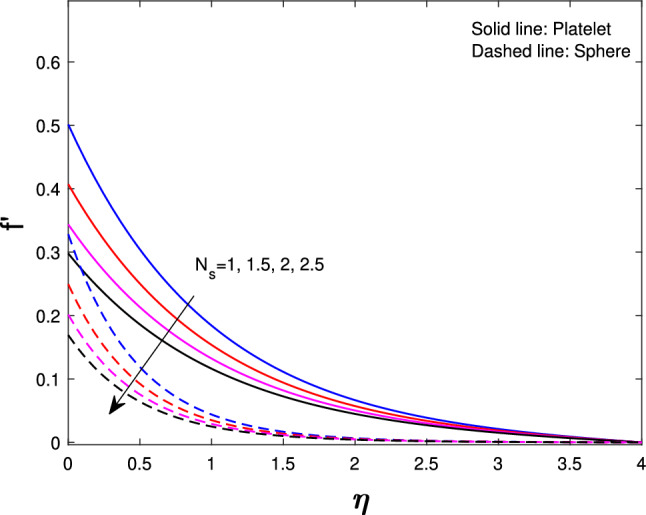
Influence of $N_{s}$ on $f^{'}{(\eta )}$

**Fig. 7:**
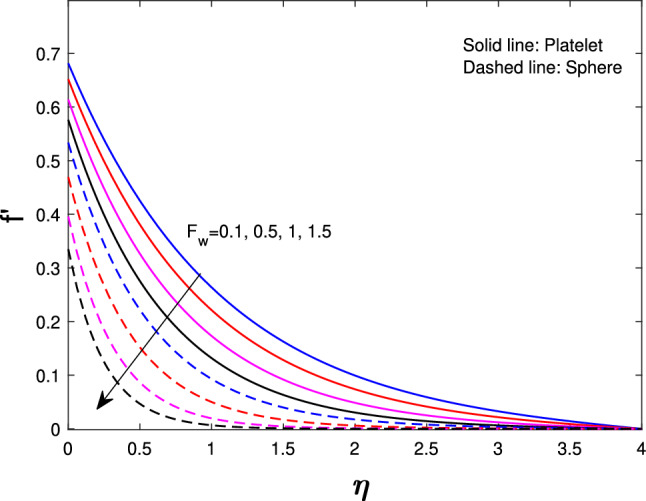
Influence of $F_{w}$ on $f^{'}{(\eta )}$

**Fig. 8:**
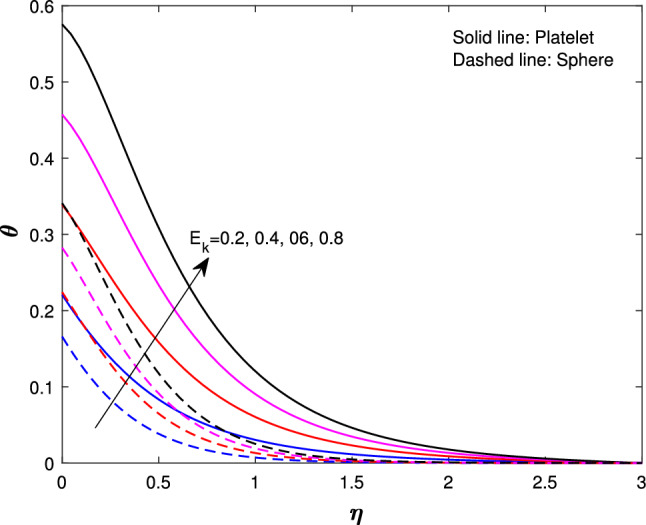
Influence of $E_{k}$ on $\theta {(\eta )}$

**Fig. 9:**
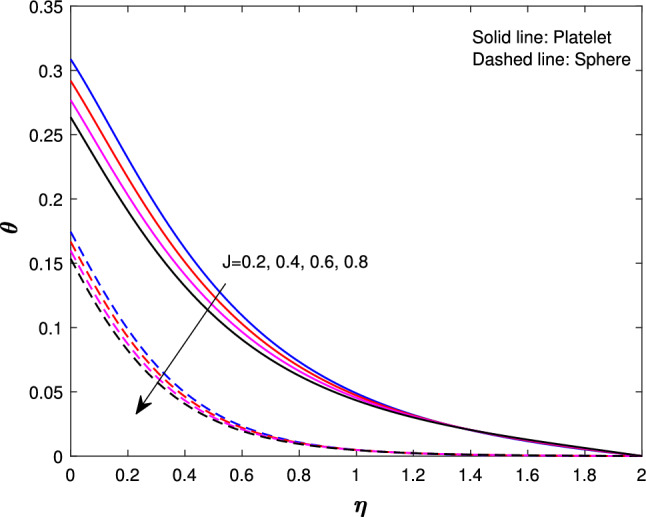
Influence of *J* on $\theta {(\eta )}$

**Fig. 10:**
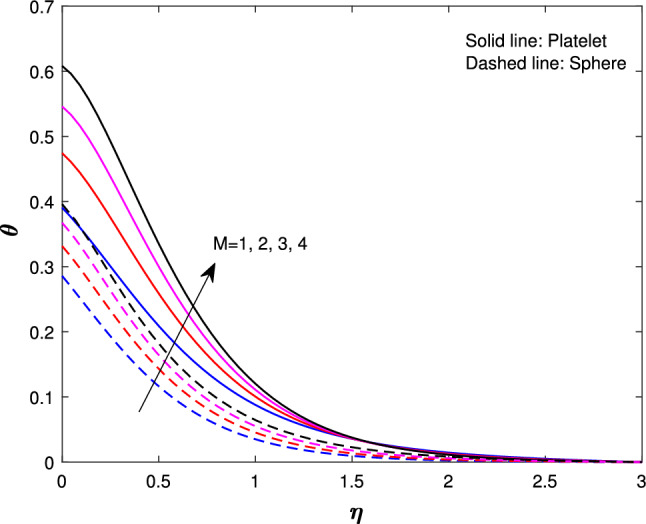
Influence of *M* on $\theta {(\eta )}$

**Fig. 11:**
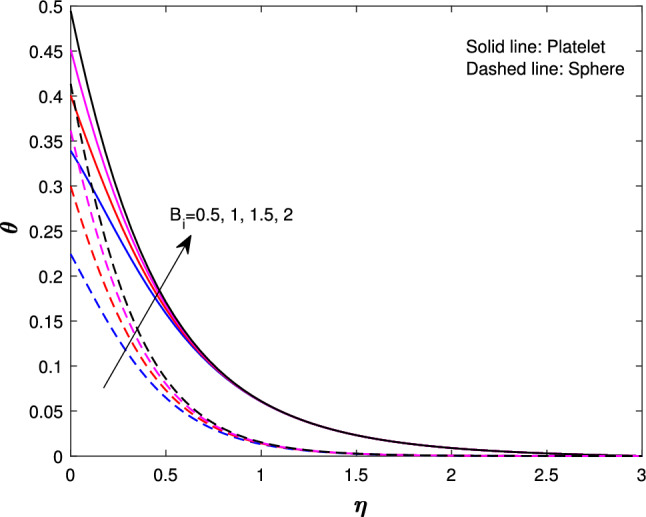
Influence of $B_{i}$ on $\theta {(\eta )}$

**Fig. 12:**
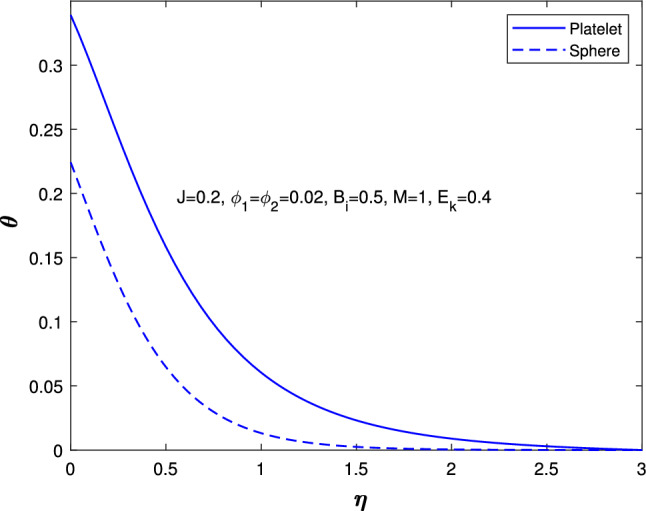
Infuence in temperature profile

Extensive numerical simulations can be executed for many possible values of non-dimensional controlling parameters, namely volume fraction $(\delta )$, unsteadiness parameter (*J*), porosity parameter $(P_{k})$, magnetic parameter (*M*), suction parameter $(F_{w})$, partial slip parameter $(N_{s})$, Eckert number $(E_{k})$, and Biot number $(B_{i})$ on velocity and temperature profile for sphere and platelet shapes of *Ag* and $(Al_{2}O_{3})$ nanoparticles the outcomes are shown graphically in Figs. 2, 3, 4, 5, 6, 7, 8, 9, 10, 11, 12. In addition, Nusselt and skin friction coefficient are examined and tabulated. For the entire research, the Prandtl number $(P_{r})$ is taken as 6. Table 1 contains the hybrid nanoparticles and base fluid thermophysical properties. Table 2 demonstrates the viscosity coefficients, shapes, and sizes of $Ag-Al_{2}O_{3}$ nanoparticles.

Fig. 2 shows the effects of nanoparticle shapes on the velocity distribution when all the other parameters $\delta _{1},\delta _{2}$=0.02, *J*=0.4, $P_{k}$=$N_{s}$=$B_{i}$=$E_{k}$=$F_{w}$=0.5, *M*=1, $P_{r}=6$ are fixed. It describes that hybrid nanoparticles in the platelet form have a high velocity whereas those in the spherical shape have a low velocity. Figure 3 indices the effects of magnetic parameter (*M*) it varies from 0 to 3 on velocity while others are fixed parameters. It shows when we increase the Magnetic parameter the sphere and platelet shape velocity profile decrease. This is because increasing the magnetic field produces the opposing force to the flow, known as the Lorentz force. This force reduces the velocity near the sheet’s surface.

Figure 4 shows the unsteadiness parameter (*J*) influence on velocity. Less heat is transported from the stretched sheet to the nanofluid in the boundary layer region so the stretched sheet’s velocity decrease for both sphere and platelet shapes as the unsteadiness parameter increase. From Fig. 5 it is observed that if we increase the porosity parameter $(P_{k})$ of a fluid that decreases velocity. As fluid flows through a porous medium, its velocity is gradually reduced because of the increased resistance to its flow. Figure 6 indicates that with the increase in slip parameter $(N_{s})$ the velocity will decrease gradually for both sphere and platelet shapes. This decrease happens because the fluid velocity close to the sheet is different from the velocity of the stretched sheet.

Figure 7 shows the effects of a suction parameter $(F_{w})$ on $f^{'}(\eta )$. These figures demonstrate that when the suction parameter is increased, velocity drastically drops for both shapes. This is consistent with the physical behavior of the suction parameter in general. Figure 8 illustrates the variation of Eckert number ${(E_{k})}$ on the temperature of sphere and platelet shapes. By exerting effort against the strains of a viscous fluid, a rise in the Eckert number promotes the process of kinetic energy turning into internal energy therefore, raising the Eckert number raises the nanofluid temperature. Figure 9 implicit the variation of *J* on temperature profile. It has been observed that increasing the unsteadiness parameter results in a significant drops in thermal profiles. This is because increasing unsteadiness enhances heat loss due to sheet stretching, resulting in a lower temperature. This means that the cooling rate is much faster for unsteady flow compared to steady flow. The reason is that for higher values of the unsteady parameter, the rate of heat transfer from the sheet to the fluid decreases, which leads to a faster cooling rate.

Figure 10 describe the magnetic parameter influence on temperature profile. An increase in the magnetic parameter could lead to greater energy confinement or changes in heat transfer mechanisms, which can result in higher temperatures throughout the system. This interaction between magnetic fields and thermal behavior is important in fields like astrophysics, plasma physics, and materials science. Figure 11 shows the effect of Biot number on the temperature profile. An increase in the Biot value causes more convection, which raises surface temperatures and increases wall temperature values. This thickens the thermal boundary layer and alters the temperature profile. so, the temperature increases for both sphere and platelet shapes. The Biot number can optimize heat exchanger and food processing design. Figure 12 describes the variation in the temperature profiles of spheres and platelets while all the parameters are fixed. We found that platelet shapes exhibit high heat transfer compared to sphere shapes.

**Fig. 13:**
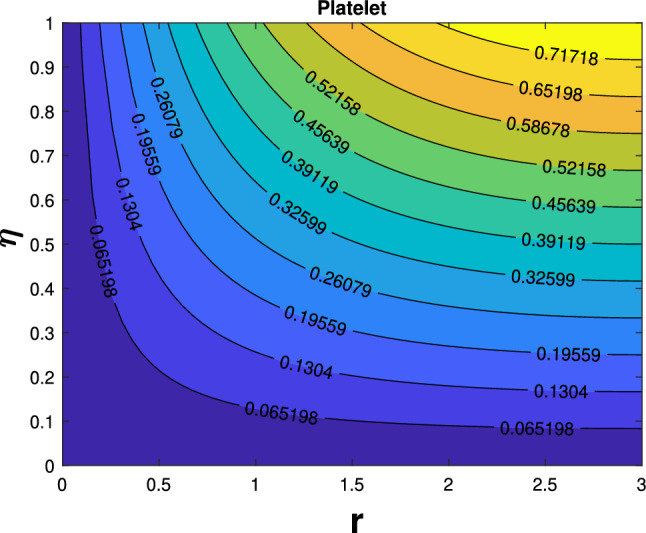
Streamline patterns of platelet shape when $M=0.0$

**Fig. 14:**
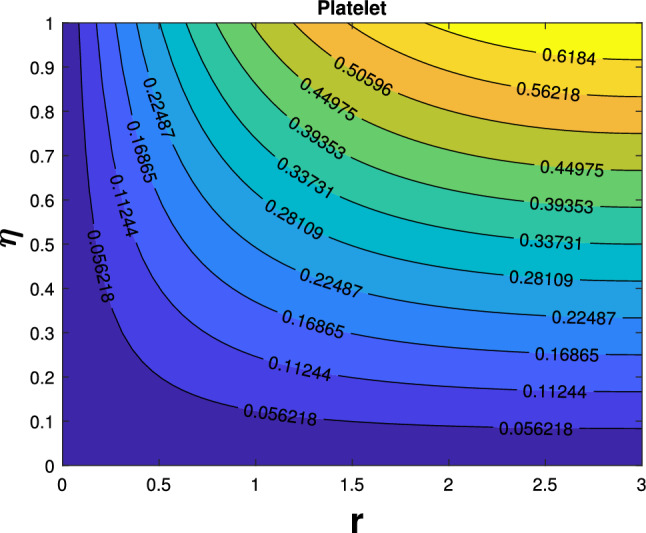
Streamline patterns of platelet shape when $M=1$

Table 3 compares the current findings to earlier findings for different values of slip. Excellent agreement exists between the current findings and the past outcomes. The impact of governing factors on the skin friction coefficient can be observed in Table 4. The table shows that an increase in the parameters like magnetic (*M*), unsteadiness (*J*), porosity $(P_{k})$, suction $(F_{w})$, and slip $(N_{s})$ minimizes the friction factor. Table 5 shows the numerical outcomes of Nusselt numbers. Rise in the values of Eckert number, magnetic parameter, Biot number escalating the Nusselt number. Heat transfer enhancement of different shapes are presented in Table 6. In overall heat transfer, platelet-shaped nanoparticle possesses high enhancement when compared to sphere-shaped nanoparticles. Figure 13 depicted platelet shaped nanoparticles streamline impacts when M = 0.0, $\delta _{1}=\delta _{2}$=0.02. Figure 14 shows streamline effects of platelet shapes at $M=1$.

**Table 1:** Thermophysical characteristics of nanoparticles and base fluid [42]

Physical characters	*Ag*	$Al_{2}O_{3}$	Water
$K (\mathrm{W/m K})$	429	40	0.613
$C_{p}{(\mathrm{J/kg K})}$	235	765	4179
$\sigma (S/m)$	62.1$\times$ $10^{6}$	35$\times$ $10^{6}$	5.5$\times$ $10^{6}$
$\rho (\mathrm{kg/m}^{3})$	10,500	3970	997.1

**Table 2:** The viscosity coefficients and size of nanoparticles [43]

Shapes	Sphere sphere1	Platelet platelet1
$w_{1}$	2.5	37.1
$w_{2}$	6.5	612.6
n	3.0	5.72

**Table 3:** Comparison of ${f^{''}(0)}$ for Various values of slip parameter $N_{s}$

$N_{s}$	Ali et al. [37]	Hayat et al. [35]	Present
0	1.1737	1.1737	1.173734
0.01	1.1534	1.1534	1.153485
0.05	1.0799	1.0799	1.079964
0.1	1.0018	1.0018	1.001850
0.5	0.6505	0.6505	0.650555
1	0.4625	0.4625	0.462547
2	0.2990	0.2990	0.299099

**Table 4:** Numerical values for skin friction

Parameters					$-C_{f}Re^\frac{1}{2}$	
*M*	$P_{k}$	$N_{s}$	$F_{w}$	*J*	Sphere	Platelet
0	0.5	0.5	0.4	0.4	1.057645	2.421580
1					1.140530	2.689875
2					1.202924	2.904920
3					1.252695	3.083574
1	0.1				1.104683	2.571224
	0.5				1.140530	2.689875
	1				1.179208	2.821968
	1.5				1.212690	2.939494
	0.5	1			0.744139	1.969050
		1.5			0.554376	1.560801
		2			0.442338	1.295719
		2.5			0.368198	1.108936
		0.5	0.1		1.033391	2.515035
			0.5		1.175643	2.749149
			1		1.338837	3.049177
			1.5		1.473526	3.347632
			0.4	0.5	1.146099	2.709425
				1.5	1.197431	2.891641
				2.5	1.241456	3.051567
				3.5	1.279410	3.192460

**Table 5:** Numerical values for nusselt number

Parameters				$NuRe^\frac{-1}{2}$	
*J*	*M*	$E_k$	$B_i$	Sphere	Platelet
0.2	0.5	0.4	0.5	0.463523	0.426287
0.4				0.467842	0.436648
0.6				0.471738	0.445890
0.8				0.475260	0.454141
0.4	1			0.400962	0.375726
	2			0.375215	0.324127
	3			0.355221	0.279995
	4			0.339100	0.241618
	1	0.2		0.468251	0.480444
		0.4		0.435515	0.407535
		0.6		0.402780	0.334625
		0.8		0.370044	0.261715
		0.4	0.5	0.435515	0.407535
			1	0.786413	0.739246
			1.5	1.075169	1.014494
			2	1.316949	1.246564

**Table 6:** Nusselt number and heat transfer enhancement

	Nu		$E_{R}$	
$\delta$	Sphere	Platelet	Sphere	Platelet
1%	0.475287	0.487027	2.74%	5.27%
2%	0.488198	0.512095	5.53%	10.69%
3%	0.501364	0.537822	8.37%	16.25%
4%	0.514788	0.564203	11.28%	21.96%
5%	0.528474	0.591235	14.23%	27.8%

## Conclusions

This research presents the shape effects of Ag-Al$_{2}$O$_{3}$ nanoparticles on unsteady flow characteristics across a radially extended surface embedded in a porous medium with convective boundary conditions, slip, suction and joule heating. After the similarity transformations are applied, the differential equations have no dimensions. Using the bvp4c solver, the nonlinear ODEs are solved numerically. Graphs illustrate the effects of several factors on temperature and velocity profiles. The future goal of this research is to understand how nanoparticle morphologies can be used for targeted drug delivery systems as well as innovative materials with enhanced properties. The following are the results of the current investigation:

- The velocity profile decrease for both shapes when M is increased, and when the unsteadiness parameter value is increased, the velocity and temperature decrease.
- A rise in the porosity, slip, and suction parameter values makes the velocity profile decrease gradually.
- The temperature profile increases when Biot number, magnetic parameter, and Eckert number increase.
- The outcomes show that platelet-shaped nanoparticles possess high heat transfer and high-velocity flow when compared to sphere-shaped nanoparticles. This is due to the greater surface area of platelet-shaped nanoparticles, which facilitates more effective heat transmission.
- The results show that the heat transfer enhancement of platelet-shaped nanoparticles improved by 11.88% compared to sphere-shaped nanoparticles while utilizing Ag-Al$_2$O$_3$/H$_2$O hybrid nanofluid with a volume fraction of 5%.
- As a result, the size and shape of the nanoparticles have a significant effect on the heat transmission of hybrid nanofluid over a radially stretching sheet.

## Data Availability

On reasonable request, the corresponding author will provide access to the datasets used and analyzed during the present work.

## List of symbols

$C_{p}$: Specific heat at constant pressure
Nu: Nusselt number
$$T_{r}$$: Reference temperature
$C_{f}$: Skin friction coefficient
$$\alpha$$: Thermal diffusivity
$f(\eta )$: Dimensionless stream function
k: Thermal conductivity
$$F_{w}$$: Suction parameter
b: Positive constant
$$N_{s}$$: Slip parameter
$\mu$: Coefficient of viscosity
*J*: Unsteadiness parameter
$$\rho$$: Fluid density
$E_R$: Rate of heat transfer enhancement
$$\sigma$$: Electrical conductivity
$$P_{k}$$: Porosity parameter
$T_{w}$: Temperature near the surface
$$B_{i}$$: Biot number
$$T_{\infty }$$: Ambient temperature
$$P_{r}$$: Prandtl number
$B_{0}$: Applied magnetic field strength
M: Magnetic parameter
*c*: Stretching rate
$$E_{k}$$: Eckert number
u,w: Velocity components along r and z directions
$$w_{0}$$: Suction velocity
$$\theta (\eta )$$: Dimensionless temperature
$U_{w}$: Velocity near the sheet
$f^{'}(\eta )$: Dimensionless velocity function
